# Non-Pharmacological approach for the management of gastroesophageal reflux disease

**DOI:** 10.12669/pjms.40.3.7291

**Published:** 2024

**Authors:** Mohammad Akram Randhawa, Sadia Azam Khan, Aqsa Naseer, Muhammad Tariq Baqai

**Affiliations:** 1 Mohammad Akram Randhawa, MBBS, PhD. Professor, Department of Pharmacology and Therapeutics, Rawalpindi Medical University, Rawalpindi, Pakistan; 2 Sadia Azam Khan, MBBS, MRCGP. Assistant Professor, Head of Department of Family Medicine, Rawalpindi Medical University, Rawalpindi, Pakistan; 3 Aqsa Naseer, MBBS, FCPS. Senior Registrar, Gastroenterology Unit, Department of Medicine, Rawalpindi Medical University, Rawalpindi, Pakistan; 4 Muhammad Tariq Baqai. MBBS, FRCP. Professor (Retired), Department of Medicine, HBS Medical and Dental College, Islamabad, Pakistan

**Keywords:** Severe GERD, Akram’s life-style, Two meals a day, Only fluids in between

## Abstract

Treatment of Gastro-Esophageal Reflux Disease (GERD) is becoming a challenge. Proton pump inhibitors (PPIs) are commonly prescribed but have many risks, particularly in long-term use. In an earlier pilot study, we have reported benefits of short-term practice of a new life-style, two meals a day with only liquids in between, for management of GERD. Present case report demonstrates benefits of long-term practice of this dietary regimen. A 61 year old patient complaining of night refluxes was diagnosed to have severe GERD with ulcerations at gastroesophageal junction and was advised to take two meals a day with only water, fruit juice, tea, or milk in intervening period. His reflux symptoms improved within fortnight and he remained well for long-time. Endoscopy done after seven years revealed competent and clear gastroesophageal junction. It is concluded that suggested life-style, “Akram’s life-style”, for GERD is a useful alternate to risky medical and surgical interventions.

## INTRODUCTION

GERD is becoming a challenge for medical profession because its prevalence is increasing, requires long-term risky drug-therapy and/or surgical interventions. Moreover, the recurrence rate is high with any mode of treatment. The worldwide prevalence of GERD was found to be 13.98%, varying from 4.16% in China to 22.4% in Turkey.^1^ Later, in a study in South Punjab, Pakistan, prevalence rate was found to be 26.6%.^2^ A Japanese study reported that irregular dietary habits had the strongest association with GERD, and PPIs in usual doses could not control the disease.^3^ PPIs are generally safe and given as first-line drugs for management of GERD. But their prolonged use, which is mostly required, causes severe achlorhydria leading to serious infections such as diarrhea and pneumonia. Their long-term use also causes deficiency of Vitamin B_12_ and minerals (such as iron, calcium and magnesium), which are linked to anemia, osteoporosis, spontaneous fractures and cardiac arrhythmia.^4^ Therefore, alternate remedies are mandatory for treatment of GERD.

Considering the pathophysiology of GERD and its strong association with irregular diet, a new life-style change, “Akram’s life-style”, i.e., two regular meals a day, morning and evening, with only liquids during the intervening period (Water, fruit juice, tea and milk, etc.), whenever patient feels hungry or thirsty, has been suggested as an alternate for management of GERD. Earlier, we have reported a pilot study of 20 patients endoscopically diagnosed to suffer from mild to severe GERD. They followed the suggested dietary regimen for two weeks and were allowed only antacids in case of urgent need, i.e., epigastric pain and reflux. 15 (75%) were free of reflux symptoms after two weeks, while two (10%) reported partial benefit.^5^

One of the participants of above mentioned pilot study continued the suggested dietary regimen for many years and remained free of symptoms without medication. Rarely, when he took solid food between meals suffered from reflux symptoms, which were relieved with antacids in a day or two. PPIs or H-2 antagonists were not used throughout follow up period, almost seven years. After seven years his endoscopy was done again to see the prognosis and is being presented.

## CASE REPORT

**Fig.1:**
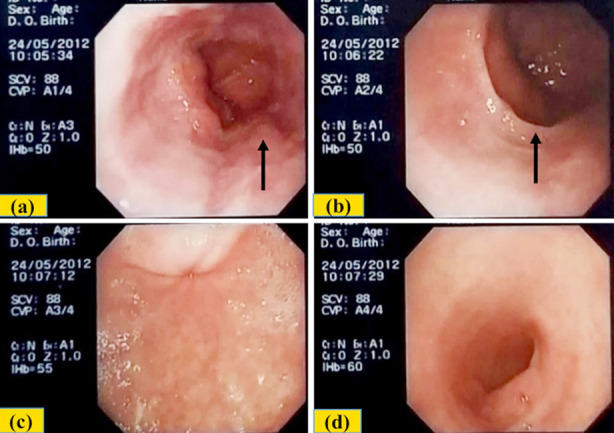
(a) Inflammation and erosions at gastroesophageal junction and a small Barrett’s (Arrow). (b) Incomplete Schatzki’s ring (Arrow). (c) Moderate diffuse erythema with mild erosions in the antrum. (d) Pylorus and initial part of duodenum appear normal.

A 61 year old man suffered from episodes of night refluxes with heart burn and sore throat for two to three months. He was taking breakfast at 6-7am, lunch at 3-4 pm, dinner at about nine pm and then sleep about one hour after dinner. Endoscopy done on May 24, 2012 showed erythema with erosions and ulcers in lower esophagus, incompetent lower esophageal sphincter and a small (3-4 mm) Barrett’s. Moreover, a partial Schatzki’s ring at gastroesophageal junction, small sliding hiatus hernia and mild erythema and erosions in the antrum were also observed. The pylorus and initial part of the duodenum were normal (Fig.1). Histopathology demonstrated mild to moderate esophagitis at gastroesophageal junction and inflammation of the antrum without *H. pylori*, atrophy or metaplasia. A diagnosis of esophagitis with erosions, ulcerations, hiatus hernia, Barrett’s and Schatzki’s ring was made (Fig.1).

**Fig.2:**
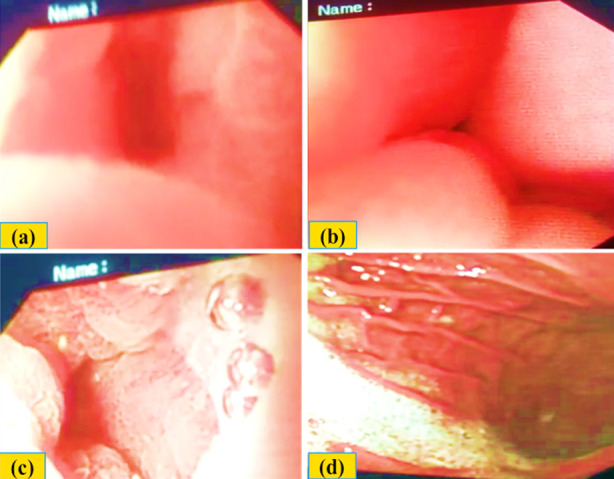
(a & b) Normal gastroesophageal junction. (c) Erosions in antrum. (d) Normal pylorus.

On March 5, 2019 his endoscopy was done again to see the prognosis, which showed competent lower esophageal sphincter without erosions or ulceration in lower esophagus. However, there were benign erosions in the stomach body and antrum. The pylorus and pyloric sphincter were normal (Fig.2).

## DISCUSSION

Irregular dietary habits are reported to have the strongest association with GERD.^3^ Because intake of meal when stomach already has food causes another surge in gastric acid secretion and distention of upper stomach, which generate Transient Lower Esophageal Sphincter Relaxations (TLESRs). TLESRs are important cause of gastric reflux. Moreover, a meal during fasting prevents development of Migrating Myoelectric Complexes (MMCs), which clear the stomach of debris and propel food onwards. Poverty of MMCs delays gastric emptying and favors reflux.^6^

‘Akram’s life-style’ for prevention and treatment of GERD involves two meals a day with only liquids in between. Two meals a day, morning and evening, allow suitable inter-digestive period and generation of MMCs, which favor gastric emptying. Intake of food when stomach and upper intestines are empty is least likely to induce reflux. Liquids during interval between meals are unlikely to delay gastric emptying or trigger reflux. Because liquid meal leaves stomach fast, transit half-time is 10 to 30 minutes. While solid diet is slow to leave, transit half-time is two to three hours.^7^

Schatzki’s ring seen at gastroesophageal junction of our patient is a benign narrow ring of tissue which develops in response to acid reflux. A large Schatzki’s ring can cause dysphagia. Whereas, Barrett’s esophagus is development of columnar epithelium and may present as small nodule at the junction of esophagus and stomach. Persistent Barrett’s over the years can lead to neoplasm.^8^ Gastric erosions are a common finding in elderly persons, usually associated with stress or some drugs (such as steroids and NSAIDS). Overall malignancy rate was lower (5%) in persons having erosions than controls (15%).^9^

Long-term use of PPIs, which is mostly required for the management of GERD, causes complications, e.g., diarrhea, pneumonia, anemia, osteoporosis and cardiac arrhythmia. Therefore, life-style interventions, e.g., weight-loss, sleeping three hours after meals, raising head side of bed while sleeping and avoidance of foods that trigger reflux (chocolates, coffee, smoking and alcohol, etc.) should be encouraged, which can minimize episodes of GERD.^10^ Our suggested life-style, two meals a day (morning and evening) with only fluids in between, provides an opportunity to take evening meal a bit earlier, i.e., 2-3 hours before going to sleep. Moreover, patient was advised to keep head-side of the bed a bit raised (with two pillows). These factors are expected to provide additional benefit to the patient.

### Limitations of the study:

An important limitation to our life-style intervention is that most people are used to take three meals a day, or some-times 4 meals a day, e.g., break-fast, lunch, dinner; or in 2^nd^ case, break-fast, lunch, evening tea with snacks and dinner. However, when they start practicing two meals a day with only fluids in between, within a week they appreciate the benefits of our dietary regimen and continue with that. In a month or two they are completely free of symptoms. To ensure better compliance involves the role of the physician, who can encourage patients for adherence to the suggested dietary regimen. Moreover, it is human nature that when one benefits from some treatment would adhere to it.

Recently, we have presented the data of 60 patients, clinically diagnosed of GERD who practiced our life-style, in World Clinical Pharmacology (WCP), 2-7 July 2023, Glasgow, UK; arranged by the British Pharmacological Society and the International Union of Pharmacologists (IUPHAR). The data showed highly significant improvement in GERD symptoms (P=0.000) after four weeks of the intervention when measured by Visual Analog Scales and 69 to 89% improvement in symptoms (Heart burn, reflux, nausea, vomiting and dyspepsia) when estimated by Yes/No response.^11^

Akram’s life-style, i.e., two regular meals a day and consumption of only liquids in between, avoids hyperacidity induced by meal on top of meal, allows MMC of inter-digestive phase to work smoothly and prevents delay in gastric emptying. Therefore, it works like anti-secretory and prokinetic drugs.

## CONCLUSIONS

Prevalence of GERD is increasing due to changes in dietary habits and its drug and surgical treatments are risky and followed by recurrence. Hopefully, ‘Akram’s life-style’, two meals a day with only fluids in between, would serve as an alternate to risky medical and surgical interventions.

### How study adds to medical literature and influences on clinical practice:

The work promotes a new life-style (Akram’s life-style) change, two meals a day with only fluids in between, for the management of Gastro-Esophageal Reflux Disease (GERD). The practice of the new life-style would avoid the use of Proton Pump Inhibitors (PPIs) and other risky medical and surgical interventions and prevent the recurrence of GERD.

### Authors’ Contribution:

**MAR:** Contributed in the concept and design of the study and in writing the initial draft of the manuscript. Also arranged the 1^st^ endoscopy for diagnosis of GERD in the case being reported. He is responsible and accountable for the accuracy and integrity of the work.

**SAK:** Contributed in follow-up, record keeping and re-writing the article, particularly the discussion.

**AN:** Contributed in reviewing the article, particularly introduction and the results of endoscopy and histology. Also updated the references.

**MTB:** Performed the 2^nd^ endoscopy, after seven years of the follow-up, to see the prognosis. Also reviewed the paper and made useful additions and alterations.
